# A Core Outcome Set to Guide Future Research on Caesarean Scar Ectopic Pregnancy: COSCAR Consensus Study

**DOI:** 10.1111/1471-0528.70090

**Published:** 2025-11-27

**Authors:** Simrit Nijjar, Ilan E. Timor‐Tritsch, Andrea Kaelin Agten, Jin Li, Krystle Y. Chong, Munira Oza, Rosanna Acklom, Francesco D'antonio, Lan N. Vuong, Ben Mol, Cecilia Bottomley, Davor Jurkovic

**Affiliations:** ^1^ Faculty of Population Health Sciences, EGA Institute for Women's Health University College London London UK; ^2^ Icahn School of Medicine at Mount Sinai New York New York USA; ^3^ Liverpool Women's Hospital NHS Foundation Trust Liverpool UK; ^4^ Department of Obstetrics and Gynaecology, College of Medicine and Health Siences UAE University Abu Dhabi UAE; ^5^ Department of Obstetrics and Gynecology, National Clinical Research Center for Obstetric and Gynecologic Diseases Peking Union Medical College Hospital, Chinese Academy of Medical Sciences and Peking Union Medical College Beijing China; ^6^ Department of Obstetrics and Gynaecology Monash University Clayton Australia; ^7^ The Ectopic Pregnancy Trust London UK; ^8^ Department of Obstetrics and Gynecology, Center for Fetal Care and High‐Risk Pregnancy University Hospital of Chieti Chieti Italy; ^9^ Department of Obstetrics and Gynecology University of Medicine and Pharmacy at Ho Chi Minh City Ho Chi Minh City Vietnam; ^10^ Monash Women's Monash Health Clayton Australia

**Keywords:** caesarean scar, consensus development study, core outcome set development, Delphi, ectopic pregnancy, stakeholder engagement

## Abstract

**Objective:**

To develop a core outcome set (COS) for research on caesarean scar ectopic pregnancy (CSEP) treatment.

**Design:**

Consensus development study.

**Setting:**

International.

**Population:**

Healthcare professionals, researchers, patient advocates, or individuals with lived experience of CSEP.

**Methods:**

The Delphi process was conducted from July to November 2024 following a systematic review and interviews with individuals with lived experience of CSEP, which identified potential outcomes. This process followed COMET guidance. Outcomes were presented in a two‐round online Delphi survey. Round one included 287 healthcare professionals, 50 researchers, and 69 patient advocates and individuals with lived experience from 51 countries. In round two, 281 participants from 43 countries contributed. The final COS was developed at a consensus meeting of the steering committee, comprising all key stakeholders.

**Results:**

346 outcomes were initially identified, reduced to 62 in round one, then 40 in round two of the Delphi survey. The final COS comprises 19 core outcomes across seven domains: (1) Treatment success in early CSEP; (2) Complications in early treated CSEP; (3) Success of expectantly managed advanced live CSEP; (4) Complications of expectantly managed advanced live CSEP; (5) Mortality and severe morbidity; (6) Future reproductive health; and (7) Patient experience. Additionally, 15 non‐mandatory outcomes and four essential reporting items were recommended. Clear definitions were provided for each core outcome.

**Conclusion:**

Through international consensus, we have developed a COS that reflects the perspectives of healthcare professionals, researchers, and individuals with lived experience of CSEP. This COS allows for standardised outcome reporting in future research, fostering a new generation of high‐quality evidence that can inform clinical guidelines and ultimately improve patient outcomes.

## 1 Introduction

With the global rise in caesarean delivery (CD) rates [1], caesarean scar ectopic pregnancy (CSEP), once rare, is now the most common type of uterine ectopic pregnancy. The impact of CSEP on future fertility, outcomes in subsequent pregnancies, and complications during ongoing CSEP pregnancies remains poorly understood, with no consensus on the optimal treatment approach [2].

Treatment options for CSEP are highly varied, with considerable differences in reported success and complication rates [3, 4]. CSEP is characterised by implantation of the gestational sac within a myometrial defect in the lower segment caesarean scar. Diagnosis can be technically challenging and result in delay or misdiagnosis, as the pregnancy may protrude predominantly into the uterine cavity, giving the appearance of a normally sited intrauterine pregnancy. Both diagnostic delays and the abnormal anatomical implantation near deeper uterine vessels contribute to a significant risk of haemorrhage and serious maternal morbidity [5, 6]. Major treatment approaches include surgical options such as suction curettage or resection through various routes, medical management with methotrexate, and other interventions like uterine artery embolisation. Choosing the correct treatment is thus challenging, but preserving fertility and patient safety should be a central consideration for any treatment approach. The existing literature on CSEP treatment is heterogeneous, with studies reporting varied outcomes, making direct comparisons and meta‐analysis impossible [2]. Furthermore, few studies report patient‐centred outcomes, which results in a critical gap in understanding the full impact of treatments. As a result, the question of the most optimal treatment for CSEP remains unanswered.

In response, we established COSCAR (Core Outcomes for Caesarean Scar Ectopic Pregnancy Research), an international collaboration of healthcare professionals, researchers, patient advocates, and individuals with lived experience of CSEP. The goal of COSCAR was to develop a consensus‐driven core outcome set (COS) for future CSEP treatment studies. Standardising outcome selection and reporting is crucial to minimising selective outcome reporting bias, where only positive or interesting outcomes are published, excluding negative findings that might challenge prevailing research narratives.

Although a COS for ‘Ectopic Pregnancy’ was published in 2023 [7], we chose to proceed with COSCAR, as the existing COS was not fully applicable to CSEP. CSEP differs from other ectopic pregnancies in its aetiology, natural progression, and associated morbidity. Compared to other ectopic pregnancies, live CSEP is much more likely to progress to viability, albeit with significant risks to the mother and the fetus. Given the potential for pregnancy continuation, a CSEP‐specific COS must address outcomes beyond the first trimester, including maternal and neonatal morbidity, which were not covered in the existing COS for ectopic pregnancy.

In this paper, we describe the process through which we developed a COS—a minimum set of critical outcomes that should be reported in future CSEP research [8]. By establishing this COS, we hope to help answer the crucial question of what the optimal treatment for CSEP is and ultimately guide the development of future treatment guidelines.

## 2 Methods

### 2.1 Study Design and Participants

The study was prospectively registered with the Core Outcome Measures in Effectiveness Trials (COMET) initiative (registration no. 2903). A three‐step, mixed‐methods approach, as recommended by the COMET initiative [8] and following the recommendations of the COS‐STAP and COS‐STAR statements [9, 10] was used to develop the COS. This process included: (1) creating a comprehensive long list of potential outcomes, (2) conducting a two‐round Delphi survey, and (3) holding a consensus meeting with stakeholders to finalise the COS. Three key stakeholder groups were involved: researchers with expertise in CSEP, healthcare professionals involved in its diagnosis and management (obstetricians, gynaecologists, interventional radiologists, anaesthetists, sonographers, nurse practitioners, and psychologists), and patient representatives, including advocates and individuals (and their partners) with lived experience of CSEP. An international steering committee of 12 members was established, representing all stakeholder groups and spanning seven countries across Europe, Asia, the Middle East, Australia, and North America.

### 2.2 Developing the ‘Long List’

The process began with a comprehensive systematic review to identify outcomes currently reported in studies on CSEP treatment [2]. This review identified 326 reported outcomes across 108 studies, which were narrowed to 88 clinically relevant outcomes after grouping closely similar outcomes and removing duplicates [2]. An additional 20 outcomes were derived from individuals and partners with lived experience of CSEP who related outcomes most important to them via four qualitative semi‐structured interviews and 22 social media survey results.

In total, 108 outcomes, categorised into six domains (Morbidity & Mortality, Treatment Sequelae, Complications, Obstetric Outcomes of Expectantly Managed CSEP, and Quality of Life), were presented to the steering committee. Additionally, the steering committee reviewed the outcomes from the previously published COS for Ectopic Pregnancy [7] and selected relevant outcomes applicable to CSEP. This ensured alignment between COSCAR and the existing COS, while allowing for the inclusion of CSEP‐specific outcomes.

After in‐depth discussions, the committee refined the list, suggested possible additional outcomes, and prioritised critical outcomes, resulting in a final list of 62 outcomes. This final list was then incorporated into a modified two‐round Delphi survey for review by all stakeholders.

### 2.3 Delphi Survey

The Delphi survey was developed using REDCap, a web‐based software platform. A pilot version of the first round was tested with six individuals, and refinements were made on the basis of their feedback. To maximise global engagement, the first round of the survey was widely promoted through national and international clinical networks, the study website (www.coscar.org), and scientific meetings using QR codes and direct website links. Researchers identified via a literature search [2] were also invited. Outreach to individuals with lived experience of CSEP was conducted through a social media campaign, as well as partnerships with patient advocacy groups. Additionally, international societies and patient advocacy groups were asked to distribute the survey to their members and promote it at global conferences.

In the first round of the Delphi survey, participants were asked to assess the importance of all 62 outcomes from the ‘long list’. At the end of this round, they were invited to suggest additional outcomes, which were reviewed by the steering committee and included in the second round where appropriate. Participants who completed round 1 and provided informed consent to be contacted again were invited via email to participate in round 2. Email addresses were collected at the end of round 1 solely for this purpose. In the second round, participants reviewed only the outcomes that had not reached consensus in the first round. Although mindful of participation and barriers, the survey was conducted exclusively in English, as previous COS studies have shown low response rates with non‐English translations [7].

### 2.4 Outcome Scoring and Defining Consensus

Participants rated each outcome on a 9‐point Likert scale, where 1 indicated “extremely unimportant” and 9 indicated “extremely important.” [11] An additional option, “I can't rate the outcome because I don't know it,” was provided. To reduce measurement error, detailed explanations were provided for each point on the scale: 1 (extremely unimportant), 2 (very unimportant), 3 (unimportant), 4 (maybe unimportant), 5 (unsure if unimportant or important), 6 (maybe important), 7 (important), 8 (very important), and 9 (extremely important) [12]. Scoring followed the Grading of Recommendations Assessment, Development and Evaluation (GRADE) framework [13], scores of 1–3 represented outcomes of limited importance, scores of 4–6 represented outcomes deemed important but not critical, and scores of 7–9 represented outcomes considered critical.

Consensus thresholds were predefined. Outcomes were excluded if ≥ 70% of participants rated them as 1–3 and < 15% rated them as 7–9. Conversely, outcomes were included if ≥ 70% rated them as 7–9 and < 15% rated them as 1–3 [8]. Outcomes that reached consensus within one stakeholder group but not others were flagged for discussion at the steering committee consensus meeting [8]. Outcomes that did not reach consensus in the first round were presented again in the second round. Participants received feedback summarising how each stakeholder group scored the outcomes in the first round and were invited to adjust their ratings on the basis of this feedback.

All outcomes, including those reaching consensus and those failing to do so, were reviewed at the final steering committee consensus meeting to finalise the COS. The final consensus meeting involved the study's steering committee, comprising 11 members purposively selected prior to the start of the study on the basis of their professional expertise or lived experience with CSEP, geographic diversity, and stakeholder group representation. These individuals were not drawn from the broader Delphi participant pool but were involved throughout the project and represented all three stakeholder categories. To ensure a fair and inclusive discussion, the meeting was chaired by an independent facilitator trained in consensus methodology. All members were given equal opportunities to express their views, with clear ground rules to promote balanced participation. Outcomes were reviewed systematically, and voting was conducted using real‐time digital polling to reduce the risk of dominance by stronger voices. Final inclusion decisions were based on structured discussion followed by voting. Outcomes were categorised into three groups: (1) outcomes to be included in the final COS, (2) outcomes excluded from the COS, and (3) outcomes recommended but not mandatory for future studies. To maximise engagement and representation from all stakeholder groups and countries, the meeting was held virtually. Domains were restructured into clinically relevant categories to improve clarity and ensure the COS was both practical and feasible.

### 2.5 Data Analysis

To ensure complete data collection, all fields in the Delphi surveys were mandatory, and participants provided their email addresses to prevent duplicate entries. Survey responses were analysed both collectively and by stakeholder group (healthcare professionals, researchers, and individuals with lived experience of CSEP). This stratified analysis ensured that the perspectives of all groups were considered independently and helped to identify variations in outcome prioritisation across stakeholder types. Data were analysed and reported as proportions of agreement for each outcome. A *p*‐value < 0.05 was considered statistically significant. Statistical analyses were conducted using SPSS version 28.0.1.1 (IBM Corporation, Armonk, NY, USA) and Microsoft Excel version 2111 (Microsoft Corporation, Redmond, WA, USA).

### 2.6 Ethics

Ethical approval was obtained from the UK NHS Health Research Authority Research Ethics Committee (reference 24/LO/0190) prior to the study's commencement. Completion of the Delphi survey was considered to imply informed consent. Participants completing semi‐structured interviews provided written informed consent.

## 3 Results

### 3.1 Delphi Participant Distribution and Response Rates

**FIGURE 1:**
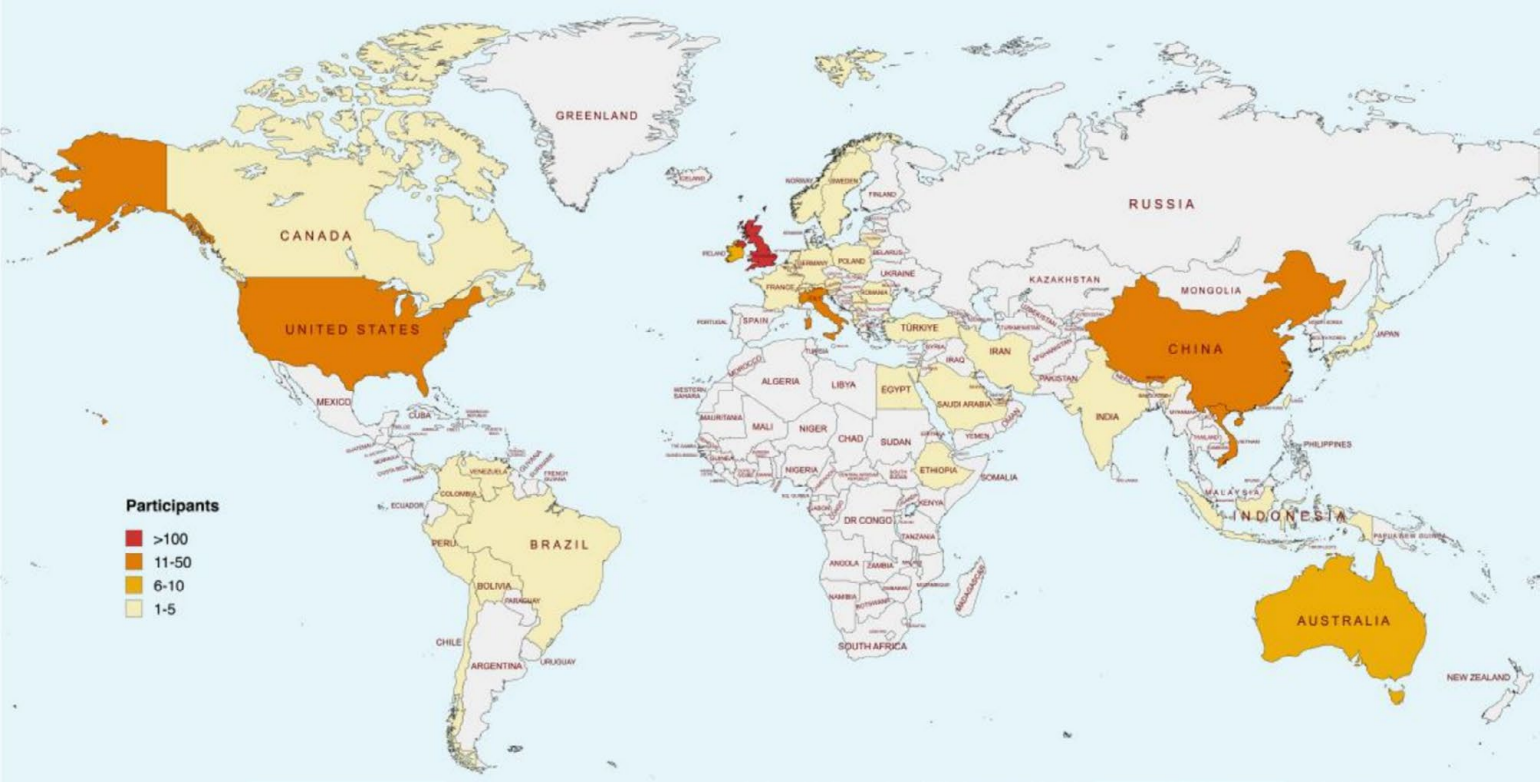
Global participant distribution: Number of participants by Country.

**FIGURE 2:**
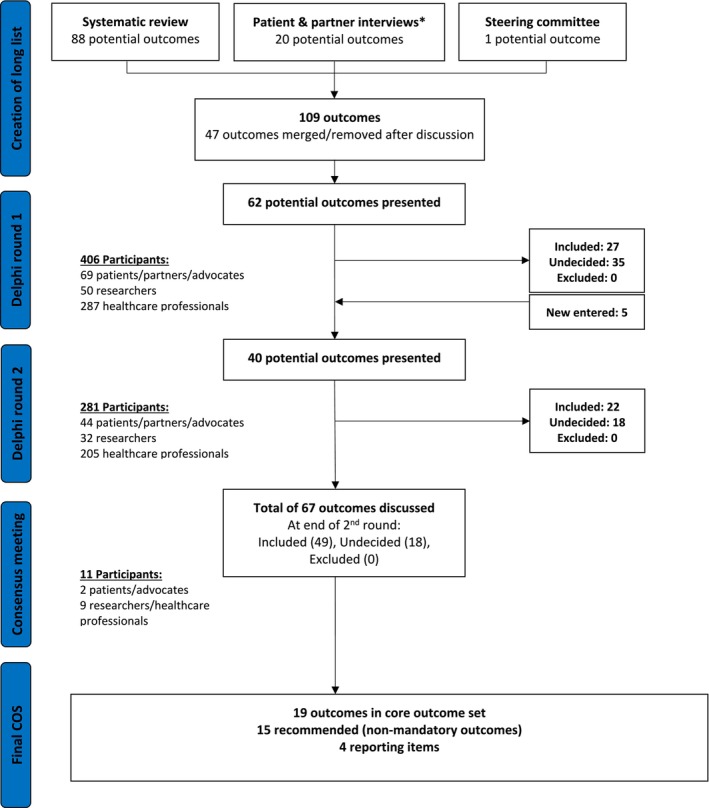
Flowchart illustrating the Delphi process for selecting potential outcomes in CSEP treatment studies. *Includes four qualitative semi‐structured interviews and 22 social media survey results.

An international consensus development study was conducted using a modified Delphi method from July to November 2024. In the first round, 62 outcomes were presented to 406 participants from 51 countries across six continents (Figure 1). Participant numbers and characteristics are described in Table 1. At the end of the first round, participants suggested five additional outcomes for consideration (Figure 2).

**TABLE 1:** Participant characteristics.

	Round 1 *n* = 406	Round 2 *n* = 281
Stakeholder group (*n*)		
Patient advocates, individuals, and partners with lived experience of CSEP	69 (17%)	44 (16%)
Researchers	50 (12%)	32 (11%)
Healthcare professionals	287 (71%)	205 (73%)
Type of post (*n*)		
Obstetrics & Gynaecology doctor	307 (91%)	217 (92%)
Nurse	7 (2%)	5 (2%)
Sonographer	7 (2%)	5 (2%)
Psychologist	2 (< 1%)	1 (< 1%)
Interventional radiologist	3 (< 1%)	4 (2%)
Other	11 (3%)	5 (2%)
Time in post (y)		
< 5	42 (12%)	21 (9%)
5–10	90 (27%)	60 (25%)
11–20	105 (31%)	83 (35%)
> 20	98 (29%)	72 (30%)
Unknown	2 (< 1%)	1 (< 1%)
Age (y)		
18–24	4 (< 1%)	1 (< 1%)
25–34	90 (22%)	52 (19%)
35–44	152 (37%)	111 (40%)
45–54	87 (21%)	63 (22%)
55–64	61 (15%)	42 (15%)
≥ 65	12 (3%)	12 (4%)
Gender (*n*)		
Female	261 (64%)	178 (63%)
Male	144 (35%)	103 (37%)
Other	1 (< 1%)	0
Geographic region (*n*)		
Africa	9 (2%)	6 (2%)
Asia	127 (31%)	64 (23%)
Central America	2 (< 1%)	1 (< 1%)
Europe	214 (53%)	174 (62%)
North America	28 (7%)	21 (7%)
Oceania	14 (3%)	7 (2%)
South America	12 (3%)	8 (3%)

In the second round, 40 outcomes were presented (five new outcomes and 35 that did not reach consensus in round 1) to 281 participants from 43 countries (Table 1) (Figure 2). By the end of the second round, 49 outcomes achieved consensus, whereas 18 outcomes remained without clear agreement. All 67 outcomes were subsequently reviewed by the COSCAR steering committee during a final consensus meeting, which included representation from all key stakeholder groups across six countries (Figure 2). Following these discussions, 19 outcomes were prioritised for inclusion in the COS for future research on CSEP treatment through consensus.

The attrition rate between rounds 1 and 2 was 31%. A Chi‐Square test showed no significant change in stakeholder group distribution between rounds, *χ* ^2^(2) = 0.42, *p* = 0.81, indicating consistent stakeholder representation throughout the Delphi process.

### 3.2 Core Outcome Set

The final COS is presented in Table 2, including those outcomes incorporated from the existing ‘Ectopic Pregnancy’ COS [7]. Detailed response rates for each outcome are available in Table S1.

**TABLE 2:** Overview of the core outcome set for studies on the treatment of CSEP, structured into core domains.

Category	Outcomes
Core outcomes (mandatory)	
Treatment success in early CSEP	Success of treatment
Complications in early treated CSEP	Complication rate Hysterectomy^a^ Blood loss^a^ Blood transfusion
Treatment success of expectantly managed advanced live CSEP	Live birth Gestational age at delivery
Complications of expectantly managed advanced live CSEP	Late pregnancy loss Obstetric hysterectomy Severe intra‐ and postpartum haemorrhage Uterine rupture
Mortality and severe morbidity	Sepsis Haemorrhagic shock Admission to ITU^a^ Mortality^a^
Future reproductive health^b^	Infertility Normally sited (eutopic) subsequent pregnancy Repeat CSEP
Patient experience	Treatment satisfaction^a^
Recommended outcomes (non‐mandatory)	
Complications in early treated CSEP	Related to non‐surgical treatments: Methotrexate adverse events^a^ Post‐embolisation syndrome Related to surgical treatments: Conversion to abdominal surgery (laparoscopy or laparotomy)^a^ Damage to surrounding structures Uterine perforation Thrombosis^a^ Retained pregnancy tissue^a^ Development of EMV (previously called AVM) following treatment
Patient experience	Psychological impact on the patient Quality of life
Recovery time	Time for serum hCG to normalise following treatment Time for retained pregnancy tissue resolution following treatment^a^ Duration of pain following treatment Duration of vaginal bleeding following treatment Time to return to normal menstruation following treatment
Reporting items	
Whether cardiac activity is present or not, if a fetus is visible Gestational age at diagnosis/treatment RMT at diagnosis and on subsequent scans (particularly for patients who are opting to continue the pregnancy) Colour Doppler assessment of pregnancy vascularity	

Abbreviations: AVM, arteriovenous malformation; CSEP, caesarean scar ectopic pregnancy; EMV, enhanced myometrial vascularity; hCG, human chorionic gonadotropin; ITU, intensive treatment unit; RMT, residual myometrial thickness.

^a^ Outcomes are also part of the existing Ectopic pregnancy COS.

^b^ These outcomes should be reported in all studies evaluating treatments for CSEP, provided the study design included women wishing for future pregnancies.

The final COS includes 19 core outcomes across seven domains (Table 2). The application of these core outcomes will vary depending on the type of CSEP treatment and the gestational age being studied. In addition, 15 non‐mandatory outcomes were recommended. The COS also outlines four essential reporting items for future studies to ensure comprehensive data collection and standardisation. The steering committee provided definitions and lay terms for each outcome to guide consistent application (Table 3).

**TABLE 3:** Comprehensive definitions and lay terms for each outcome.

Outcome (per category)		Definition	Source/Reference
Medical term	Lay term		
Treatment success in early CSEP			
Success of treatment	Whether the treatment is thought to have worked	No need for additional intervention after the planned primary treatment protocol. (All interventions should be subcategorised into: (a) As per protocol (forming part of routine treatment).^a^ (b) Out of protocol (forming part of rescue treatment)^b^).	COSCAR steering committee
Complications in early CSEP			
Complication rate:	Percentage of patients who experience problems or adverse effects as a result of treatment	Unintended consequences of the intervention, such as harm, negative effects, toxicity, complications, adverse reactions, or sequelae.	Dodd et al. [14]
Hysterectomy	Removal of the womb	Surgical removal of the uterus (when forming part of rescue treatment rather than primary treatment protocol).	COSCAR Steering Committee
Blood loss	Blood loss from treatment	The volume (ml) of blood loss during or immediately after treatment.	COSCAR Steering Committee
Blood transfusion	Need for blood transfusion	Blood transfusion required as a result of haemodynamic instability due to major blood loss.	Chong et al. [7], ectopic pregnancy core outcome set
Methotrexate adverse events	Complications directly as a result of treatment with methotrexate, which may show as abnormal function of the bone marrow, kidney/liver/blood clotting/blood cell counts	Severe adverse effects associated with methotrexate treatment which may include hepatotoxicity, pulmonary toxicity, increased risk of infections, gastrointestinal or cutaneous ulcers, myelosuppression, and nephrotoxicity.	COSCAR Steering Committee
Post‐embolisation syndrome	A constellation of signs and symptoms directly as a result of uterine artery embolisation: pelvic pain, abdominal/hip pain, nausea, vomiting, fever and complications include arterial embolism/blood clots developing in the arteries, reduced ovarian function	Post embolisation syndrome typically manifests within the first 72 h postprocedure as a collection of symptoms that may include pain, low grade fever, nausea, and vomiting, myalgia, mild leucocytosis and discharge.	Waldron et al. [15]
Conversion to abdominal surgery (laparoscopy or laparotomy)	Requiring keyhole or open surgery when this was not the initial treatment	Requiring laparoscopy or laparotomy not planned as part of the primary treatment protocol.	COSCAR Steering Committee
Damage to surrounding structures	Damage to nearby organs during or as a result of treatment i.e., the bladder, bowel, blood vessels Etc	Harm or injury caused to nearby tissues or organs during a procedure.	COSCAR Steering Committee
Uterine perforation	When a hole is made accidentally in the wall of the womb during surgery	A full thickness breach in the myometrial layer of the uterus, whether asymptomatic or resulting in damage to adjacent structures, including visceral organs and blood vessels.	COSCAR Steering Committee
Thrombosis	Blood clots that develop in the vessels in the legs or in the lungs	Formation of a thrombus (blood clot) in a deep vein, which partially or completely obstructs blood flow. The thrombus can dislodge and travel in the blood, especially to the pulmonary arteries. Thrombosis usually affects the deep veins of the legs or pelvis but may affect other sites such as the upper limbs and the intracranial and splanchnic veins.	NICE [16]
Retained pregnancy tissue	Retained pregnancy tissue following treatment	Residual trophoblastic tissue persisting following the completion of planned treatment, as confirmed by ultrasound imaging and/or histology.	COSCAR Steering Committee
Development of EMV (previously called AVM) following treatment	Formation of abnormal blood vessels with abnormal blood flow in the womb after treatment	Enhanced myometrial vascularity (EMV) is characterised by an increase in blood flow within the myometrium on colour Doppler imaging, due to retained pregnancy tissue.	COSCAR Steering Committee
Treatment success of expectantly managed advanced live CSEP			
Live birth	Delivery of a live baby	The complete expulsion or extraction from a woman of a product of fertilisation, after 24 completed weeks of gestational age, which, after such separation, breathes or shows any other evidence of life, such as heartbeat, umbilical cord pulsation, or definite movement of voluntary muscles, irrespective of whether the umbilical cord has been cut or the placenta is attached.	Modified from Duffy et al. [17], infertility core outcome set
Gestational age at delivery	At how many weeks of pregnancy the baby is born	The age of a fetus is calculated by the best obstetric estimate determined by assessments, which may include early ultrasound, and the date of the last menstrual period, and/or perinatal details. In the case of assisted reproductive techniques, it is calculated by adding 14 days to the number of completed weeks since fertilisation.	Duffy et al. [17], infertility core outcome set
Complications of expectantly managed advanced live CSEP			
Late pregnancy loss	Spontaneous loss of the baby after the first 12 weeks of pregnancy	Second trimester loss: The spontaneous loss of the pregnancy after 12 completed weeks and prior to 24 completed weeks of gestational age.	COSCAR Steering Committee
		Stillbirth: The death of a foetus prior to the complete expulsion or extraction from its mother after 24 completed weeks of gestational age. The death is determined by the fact that, after such separation, the foetus does not breathe or show any other evidence of life, such as heartbeat, umbilical cord pulsation or definite movement of voluntary muscles.	Modified from Duffy et al. [17], infertility core outcome set, RCOG, Green‐top Guideline No. 55 [18]
		Neonatal mortality: Death of a live born baby within 28 days of birth. This can be sub‐divided into early neonatal mortality, if death occurs in the first 7 days after birth and late neonatal mortality, if death occurs between 8 and 28 days after birth.	Duffy et al. [17], infertility core outcome set
Obstetric hysterectomy	Removal of the womb at the time of delivery of the baby	Surgical removal of the uterus during pregnancy or up to 42 days postpartum (planned or unplanned).	Schaap et al. [19], severe maternal morbidity core outcome set
Severe intra‐ and postpartum haemorrhage	Severe blood loss at the time and after the delivery of the baby	Refers to excessive bleeding during or within 24 h after delivery, defined as blood loss ≥ 2000 mL. It can lead to maternal morbidity.	RCOG, Green‐top Guideline No. 52 [20]
Uterine rupture	A spontaneous tear in the wall of the womb during pregnancy	A visually confirmed, complete rupture of the myometrium and serosa.	Schaap et al. [19], severe maternal morbidity core outcome set
Mortality and severe morbidity			
Sepsis	A life‐threatening condition that arises when the body's response to an infection injures its own tissues and organs, with the immune system going into overdrive	A life‐threatening organ dysfunction caused by a dysregulated host response to infection.	Singer et al. [21]
Haemorrhagic shock	Severe blood loss which can be life threatening	A life‐threatening condition caused by severe blood loss, resulting in inadequate tissue perfusion and oxygen delivery, leading to cellular and organ dysfunction. It is characterised by hypotension, tachycardia, altered mental status, and signs of organ failure.	American College of Surgeons Committee on Trauma [22]
Admission to ITU	Admission to Intensive Care Unit	Requires admission to the intensive care unit for advanced respiratory support alone or monitoring and support for 2 or more organ systems.	Intensive care society consensus statement [23], Chong et al. [7], ectopic pregnancy core outcome set
Mortality	Death	Mortality from any cause related to or aggravated by pregnancy or its management (excluding accidental or incidental causes) during pregnancy and childbirth or within 42 days of termination of pregnancy, irrespective of the duration of the pregnancy.	Modified from WHO [24]
Future reproductive health			
Infertility	Difficulty trying to conceive	Failure to achieve a successful pregnancy after 12 months or more of regular, unprotected sexual intercourse or due to an impairment of a woman's/person's capacity to reproduce either as an individual or with her/their partner.	ASRM [25]
Normally sited (eutopic) subsequent pregnancy	The next pregnancy is correctly sited (i.e., not an ectopic)	A pregnancy which is located within the uterine cavity with or without an embryo or embryonic heart pulsations.	Kirk et al. [26], ESHRE recommendations
Repeat CSEP	The next pregnancy is a caesarean scar ectopic pregnancy again	A subsequent caesarean scar ectopic pregnancy (a pregnancy implanted in the transverse lower segment caesarean section scar defect and can be classified as either partial or complete).	Kirk et al. [26], ESHRE recommendations
Patient experience			
Treatment satisfaction	Patient feeling of satisfaction with treatment	Patient satisfaction with care received including treatment and care providers.	Chong et al., 2023, ectopic pregnancy core outcome set [7]
Psychological impact on the patient	Emotional impact on the person, including anxiety, depression, post‐traumatic stress disorder or general psychological distress which does not fit into a formal diagnosis	Anxiety, depression, post‐ traumatic stress and general psychological distress in a person as a result of the CSEP. The following self‐reporting surveys have been validated in pregnancy loss populations and have been shown to have good psychometric properties. – The hospital anxiety and depression scale (HADS); – Post‐traumatic Diagnostic Scale (PDS‐5); – Clinical Outcomes in Routine Evaluation 10 (CORE‐10).	COSCAR Steering Committee Zigmond et al. [27] National Center for PTSD [28] Barkham et al. [29]
Quality of life	The person's emotional, social, and physical well‐being, and their ability to function in day‐to‐day activities	Quality of life is the individuals' perceptions of their position in life in the context of the culture and value systems in which they live and in relation to their goals, expectations, standards, and concerns.	Centers for Disease Control and Prevention, 2000 [30]

^a^ Routine treatment refers to planned interventions administered according to standard clinical protocols.

^b^ Rescue treatment refers to additional or emergency interventions used when initial treatments fail or complications develop.

### 3.3 Early Versus Advanced CSEP Outcomes: Treatment Success

Outcomes were categorised into two clinically relevant groups: early versus advanced CSEP. Treatment success was defined by consensus as: (1) absence of additional intervention after the planned primary treatment if the patient opted not to continue the pregnancy, or (2) live birth for CSEP cases where pregnancy was continued. Researchers are expected to report live birth and gestational age at delivery, the latter serving as a marker of treatment success and a predictor of neonatal and childhood health outcomes.

The inclusion of ‘treatment success’ in the COS achieved agreement among all stakeholder groups, with 92.4% overall consensus in round 1 of the Delphi survey. The outcome ‘additional intervention required’ also reached consensus in round 1 but was excluded from the final COS to avoid redundancy, as it was encompassed within the treatment success definition.

### 3.4 Early Versus Advanced CSEP Outcomes: Complications

To optimise the uptake of this COS, complication‐related outcomes were categorised into two domains on the basis of their relevance to early or advanced CSEPs.

For early CSEPs, three outcomes—hysterectomy, blood loss, and blood transfusion—were included in the COS. Hysterectomy and transfusion achieved consensus in round 1 with agreement across all stakeholder groups. Blood loss reached consensus only after re‐presentation in round 2, achieving agreement from all groups.

For advanced CSEPs, where pregnancies are continued (expectant management), four outcomes—late pregnancy loss, obstetric hysterectomy, severe haemorrhage, and uterine rupture—were included. Haemorrhage reached consensus in round 1 (87.7% overall), whereas others required re‐presentation in round 2. Following discussion in the consensus meeting, “second trimester miscarriage” was redefined as “late pregnancy loss” to encompass other types of pregnancy loss beyond miscarriage. The outcomes late pregnancy loss (initially termed second‐trimester miscarriage) and obstetric hysterectomy narrowly missed the consensus threshold in round 1 because of lower agreement among patients/partners (66.7% and 69.6%, respectively). However, when re‐presented in round 2, patient/partner agreement increased to 89% for late pregnancy loss and 93% for obstetric hysterectomy, thereby achieving consensus. Uterine rupture, introduced in round 2, achieved 94.3% overall agreement.

Placenta accreta syndrome (PAS) surpassed the agreement threshold across all stakeholder groups but was excluded following deliberation in the consensus meeting. The primary issue was the lack of a standardised definition for PAS, whether sonographic, clinico‐surgical, or histopathological. Without an agreed definition, the inclusion of PAS in the COS was deemed unfeasible as a meaningful outcome for study comparisons [31].

### 3.5 Mortality and Severe Morbidity Outcomes

All four outcomes—sepsis, haemorrhagic shock, intensive treatment unit (ITU) admission, and mortality—achieved agreement across all stakeholder groups. The steering committee confirmed their clinical relevance and inclusion in the COS.

### 3.6 Future Reproductive Health Outcomes

The steering committee recommended mandatory reporting of three outcomes—infertility, the rate of normally sited (eutopic) subsequent pregnancies, and the rate of repeat CSEP—all of which achieved agreement across all stakeholder groups.

Several other outcomes, though achieving consensus in the Delphi survey, were excluded after further discussion. These included intrauterine adhesions (IUA), the length of time until it is perceived safe to attempt conception after CSEP, and subsequent pregnancy rates on the basis of the size of the niche and the rate of caesarean scar niche repair prior to subsequent pregnancy.

The committee decided not to include IUA, as there are no universally agreed‐upon criteria for its diagnosis. Similarly, the outcome of ‘length of time until perceived safe to try to conceive’ was excluded, as the steering committee acknowledged that such outcomes are inconsistently reported and influenced by numerous external factors, such as maternal age. The committee also concluded that mandatory reporting of ‘subsequent pregnancy rates on the basis of the size of the niche’ and the ‘rate of caesarean scar niche repair prior to subsequent pregnancy’ would not be feasible for all studies.

### 3.7 Outcomes Related to Patient Experience

The patient experience encompasses more than just the physical effects or impacts of a treatment; it also includes how individuals perceive the care they receive and their interactions with healthcare providers and any psychological sequelae of the treatment. This broader perspective is captured by the outcome ‘treatment satisfaction.’ Introduced as a new item in round 2, this outcome achieved overall agreement (78.0%) among all three stakeholder groups. The steering committee agreed to include it in the COS, consistent with the existing COS for ectopic pregnancy that also included treatment satisfaction as a core outcome and recommended the Short Assessment of Patient Satisfaction (SAPS) questionnaire as the measurement tool [32]. The availability of this validated instrument further supported its inclusion in COSCAR.

Two additional outcomes, quality of life (QoL) (77.1%) and psychological impact on patients (73.2%), also reached consensus across all stakeholder groups. The steering committee acknowledged their high clinical relevance and their particular importance to patients and their partners. However, it was noted that researchers might face challenges in consistently reporting these outcomes because of the lack of consensus on validated measurement tools. As a result, these outcomes were classified as highly recommended but non‐mandatory for inclusion in the COS.

### 3.8 Non‐Mandatory Outcomes

Several outcomes presented in the Delphi survey focused on the time to resolution of clinical parameters, including time to return to normal menstruation following treatment, time for retained pregnancy tissue resolution, time for serum human chorionic gonadotropin normalisation, duration of pain, and duration of vaginal bleeding following treatment.

The steering committee decided not to include time‐related outcomes in the COS because of significant challenges in their reporting and standardisation. These outcomes are highly patient‐specific, influenced by personal factors, and are further affected by external influences such as practicalities of follow‐up schedules, concurrent treatments, and personal choices.

### 3.9 Reporting Items

It was agreed that recommending diagnostic criteria for CSEP is beyond the scope of this COS. However, the steering committee reached 100% consensus on four diagnostic reporting items, which should be included in future studies on CSEP: presence or absence of cardiac activity when a fetus is visible; gestational age at diagnosis and/or treatment; residual myometrial thickness (RMT) at diagnosis and during subsequent scans; and colour Doppler assessment of pregnancy vascularity.

## 4 Discussion

### 4.1 Main Findings

COSCAR represents a new COS to standardise the selection, collection, and reporting of outcomes in future studies investigating CSEP treatments. The COS is applicable to all treatments for CSEP, including medical, surgical, and other interventional approaches, as well as continuing the pregnancy (expectant management). It includes 34 outcomes in total, with 19 designated as core (mandatory), 15 as highly recommended but not mandatory, and four as diagnostic reporting items required for interpretation of the treatment outcomes.

### 4.2 Interpretation

The need for standardised outcome reporting in CSEP research is well‐recognised, with prior studies noting significant variability that hinders comparison and evidence synthesis [2]. Our comprehensive COS, shaped by healthcare professionals, researchers, and those with lived experience, tackles this gap. It paves the way for more consistent, robust studies, driving better evidence‐based decisions in clinical care and research.

This COS, through standardised outcome reporting, should enhance the quality of CSEP treatment research, reduce heterogeneity, and facilitate more effective synthesis of evidence through meta‐analysis. This, in turn, should benefit clinicians and patients by providing clearer guidance for patient counselling regarding treatment options. To achieve this objective, it is essential that the COS for treatment of CSEP is widely adopted to maximise meaningful research results and minimise research waste caused by poor‐quality studies. The next critical step toward achieving this goal is to define appropriate measurement tools for each outcome included in the COS.

Although several outcomes, such as PAS, IUA, and certain subsequent pregnancy metrics, achieved consensus during the Delphi survey, these were excluded from the final COS following structured discussion by the steering committee. The final consensus meeting involved representatives from all stakeholder groups—healthcare professionals, researchers, and individuals with lived experience of CSEP or patient advocates—who unanimously agreed to exclude these outcomes because of the absence of standardised definitions or diagnostic criteria. This decision was also influenced by the need to keep the COS concise and feasible, as COSCAR already includes a larger number of outcomes than are typically seen. The decision was not based on a perceived lack of importance, but rather on concerns about feasibility, inconsistent reporting, and interpretability across studies. Nonetheless, the committee recognised the clinical relevance of these outcomes and encourages their collection and reporting in future research. As measurement tools and definitions evolve, these outcomes should be reconsidered for inclusion in future updates to the COS. This approach aligns with the iterative nature of COS development and with COMET guidance, which advocates for the inclusion of patient‐important outcomes, even when measurement methods are still being refined.

To assess the uptake of the COS, we will review research registry records, published protocols, randomised controlled trials, and systematic reviews, and perform citation analyses. Monitoring the implementation of the COS will also offer insights into areas where modifications may be needed in response to emerging advancements in the CSEP field.

### 4.3 Strengths and Limitations

A robust methodological approach, guided by frameworks recommended by the COMET initiative [8], was utilised throughout the development of this COS. One of the key strengths of this COS lies in its genuinely global nature, with input from over 50 countries across six continents. Despite CSEP being a relatively rare condition, we collected over 400 responses, including contributions from 17% of individuals with lived experience of CSEP in our study. The attrition rate was 31%, which is notably lower than that of other recent COS studies, which ranged from 42%–57% [7, 33], further underscoring the robustness of our study design.

Public and patient involvement was central to this study. We ensured that individuals with lived experience of CSEP had a significant voice at every stage of the study, from the initial conceptualisation through to survey completion and participation in the steering committee. Additionally, we prioritised diversity of perspectives by including steering committee members with varying expertise and views, from a wide range of geographic locations, including non‐English speakers. This diversity helped ensure that the discussions on outcomes were well‐rounded and not one‐sided. Representation from the People's Republic of China was particularly important, given that a recent systematic review found that 83% of published research on CSEP originated from this region [2]. To enhance clarity and minimise misunderstandings, we included lay terms and definitions for each outcome, sourced from high‐quality references, including existing COS [7, 34, 35].

Despite achieving a good response rate across both rounds of the Delphi survey, there was an overrepresentation of healthcare providers and researchers compared to individuals with lived experience of CSEP.

Although QoL and psychological impact were highly prioritised by all stakeholder groups, their classification as highly recommended but non‐mandatory outcomes within the COS reflects concerns regarding the current lack of consensus on validated measurement tools. Despite broad agreement on their importance, the steering committee was unable to reach consensus on including these outcomes as mandatory within the COS. However, this approach may inadvertently deprioritise the critical importance of these outcomes and impede progress toward the development or validation of appropriate instruments. The COMET Initiative emphasises the inclusion of patient‐important outcomes in COS development, even when measurement tools are imperfect or still evolving, to ensure these domains remain central to research and clinical practice. Furthermore, a range of general health‐related quality of life (HRQoL) instruments, such as the SF‐36 and EQ‐5D, are widely available and can provide valuable interim measurement approaches. Recognising QoL and psychological impact as mandatory outcomes could incentivise efforts to refine existing tools or develop new, disease‐specific measures. Future updates to the COS should revisit the mandatory status of these outcomes as measurement science advances. Although COSCAR already includes 19 outcomes—which is greater than what is typically seen in core outcome sets—care was taken to avoid making the set overly burdensome to implement. Nonetheless, QoL and psychological impact remain critical domains and have been clearly highlighted as highly recommended outcomes, which we hope will encourage their ongoing inclusion in research.

Another limitation is that the Delphi survey was conducted online, requiring participants to have access to computers and the internet, which may have limited participation from individuals in low‐income countries. Additionally, we did not translate the survey into languages other than English, which further restricted participation from non‐English speakers. However, despite this, Vietnam and China were among the top three countries with the most survey responses.

Although we recruited participants globally, a disproportionate number of responses came from Europe, which accounted for 61.9% of the total survey participants. Conversely, response rates from low‐income regions, such as Africa, were notably low (2.1%). This may be due to the relative rarity of CSEP diagnoses, as expert ultrasound or costly MRI imaging may be less accessible in low‐resource settings.

We decided not to conduct a third round of the Delphi survey, despite 18 outcomes remaining without clear agreement following the second round. This decision was based on the belief that significant consensus was unlikely, given the complex nature of CSEP and the variability in clinical perspectives and treatment approaches. Another limitation of our study is the relatively large size of our final COS, which includes more than 10 outcomes. We recognise that larger COS may pose challenges for implementation and practical usability. Despite conducting two international Delphi rounds and engaging key stakeholders in consensus discussions, we were unable to reduce the number of outcomes further. Considering the complexity of CSEP and its diverse treatment options—which include both pregnancy termination and expectant management to full term—it was essential to retain outcomes that adequately capture the entire spectrum of treatments. As a result, we proceeded with the proposed 19 outcomes to ensure comprehensive coverage of clinically relevant endpoints.

## 5 Conclusion

This COS will contribute to higher‐quality research on CSEP treatment by standardising the selection, collection, and reporting of treatment outcomes. It will serve as a valuable tool for researchers evaluating various treatments for CSEP, including in randomised controlled trials and systematic reviews. Future research will focus on developing robust measurement tools for these outcomes to ensure consistency and comparability across studies. To maintain its relevance and accuracy, the COS will be periodically reviewed and updated in response to advancements in the diagnosis, treatment, or understanding of CSEP, ensuring that it remains a reliable and up‐to‐date framework in CSEP research.

## Author Contributions

The conception and design of this paper were conceived by the COSCAR steering committee (S.N., I.E.T.‐T., A.K.A., J.L., K.Y.C., M.O., R.A., F.D., L.N.V., B.M., C.B., and D.J.). S.N. conducted the primary analysis of the data. S.N. wrote the initial draft manuscript. All authors contributed to the interpretation of the data, revised this article critically, and agreed upon the final manuscript prior to submission.

## Funding

The authors have nothing to report.

## Conflicts of Interest

The authors declare no conflicts of interest.

## Supporting information


**Table S1:** Rating of the outcomes in the final core outcome set, by stakeholder group. Score 1–3 = 1—extremely unimportant, 2—very unimportant, 3—unimportant; Score 7–9 = 7—important, 8—very important, 9—extremely important; OC, outcome.

## Data Availability

Data available on request from the authors.
