# Supplementary material for: A Core Outcome Set to Guide Future Research on Caesarean Scar Ectopic Pregnancy: COSCAR Consensus Study
Source: BJOG. 2025 Nov 27;133(4):725–38. doi: 10.1111/1471-0528.70090 (PMC12884206; doi:10.1111/1471-0528.70090)
Supplement: Supplementary file 1 — Table S1: Rating of the outcomes in the final core outcome set, by stakeholder group. Score 1–3 = 1—extremely unimportant, 2—very unimportant, 3—unimportant; Score 7–9 = 7—important, 8—very important, 9—extremely important; OC, outcome. [file BJO-133-725-s001.docx]

**Table S1. Rating of the outcomes in the final core outcome set, by stakeholder group.**

| **Outcome** | **Rating** | **Stakeholder group** | | | |
| --- | --- | --- | --- | --- | --- |
|  |  | **Patient/partner**  **n (%)** | **Researcher**  **n (%)** | **Healthcare professional**  **n (%)** | **Total**  **n (%)** |
| **Success of treatment** | I don’t know OC | 1 (1.4) | 2 (4) | 5 (1.7) | 8 (2.0) |
|  | Scored 1-3 | 6 (8.7) | 0 (0) | 6 (2.1) | 12 (3) |
|  | Scored 7-9 | 57 (82.6) | 45 (90.0) | 273 (95.1) | 375 (92.4) |
| **Complication rate** | I don’t know OC | 1 (1.4) | 1 (2) | 4 (1.4) | 6 (1.5) |
|  | Scored 1-3 | 6 (8.7) | 0 (0) | 7 (2.4) | 13 (3.2) |
|  | Scored 7-9 | 56 (81.2) | 42 (84.0) | 259 (90.2) | 357 87.9) |
| **Hysterectomy** | I don’t know OC | 3 (4.3) | 1 (2.0) | 9 (3.1) | 13 (3.2) |
|  | Scored 1-3 | 8 (11.6) | 0 (0) | 14 (4.9) | 22 (5.4) |
|  | Scored 7-9 | 52 (75.4) | 42 (84.0) | 244 (85.0) | 338 (83.3) |
| **Blood loss** | I don’t know OC | 0 (0) | 0 (0) | 1 (0.5) | 1 (0.4) |
|  | Scored 1-3 | 3 (6.8) | 0 (0) | 5 (2.4) | 8 (2.8) |
|  | Scored 7-9 | 36 (81.8) | 29 (90.6) | 185 (90.2) | 250 (89.0) |
| **Blood transfusion** | I don’t know OC | 1 (1.4) | 1 (2) | 5 (1.7) | 7 (1.7) |
|  | Scored 1-3 | 7 (10.1) | 0 (0) | 9 (3.1) | 16 (3.9) |
|  | Scored 7-9 | 50 (72.5) | 41 (82.0) | 235 (81.9) | 326 (80.3) |
| **Live birth** | I don’t know OC | 0 (0) | 0 (0) | 1 (0.5) | 1 (0.4) |
|  | Scored 1-3 | 2 (4.5) | 0 (0) | 2 (1.0) | 4 (1.4) |
|  | Scored 7-9 | 41 (93.2) | 31 (96.9) | 198 (96.6) | 270 (96.1) |
| **Gestational age at delivery** | I don’t know OC | 0 (0) | 0 (0) | 0 (0) | 0 (0) |
|  | Scored 1-3 | 3 (6.8) | 1 (3.1) | 2 (1.0) | 6 (2.1) |
|  | Scored 7-9 | 34 (77.3) | 31 (96.9) | 195 (95.1) | 260 (92.5) |
| **Late pregnancy loss** | I don’t know OC | 0 (0) | 1 (3.1) | 1 (0.5) | 2 (0.7) |
|  | Scored 1-3 | 2 (4.5) | 0 (0) | 4 (2.0) | 6 (2.1) |
|  | Scored 7-9 | 39 (88.6) | 28 (87.5) | 185 (90.2) | 252 (89.7) |
| **Obstetric hysterectomy** | I don’t know OC | 0 (0) | 0 (0) | 1 (0.5) | 1 (0.4) |
|  | Scored 1-3 | 2 (4.5) | 0 (0) | 2 (1.0) | 4 (1.4) |
|  | Scored 7-9 | 39 (88.6) | 32 (100) | 200 (97.6) | 271 (96.4) |
| **Severe intra and postpartum haemorrhage** | I don’t know OC | 9 (13.0) | 1 (2) | 3 (1.0) | 13 (3.2) |
|  | Scored 1-3 | 7 (10.1) | 0 (0) | 7 (2.4) | 14 (3.4) |
|  | Scored 7-9 | 50 (72.5) | 44 (88.0) | 262 (91.3) | 356 (87.7) |
| **Uterine rupture** | I don’t know OC | 2 (4.5) | 0 (0) | 0 (0) | 2 (0.7) |
|  | Scored 1-3 | 2 (4.5) | 0 (0) | 2 (1.0) | 4 (1.4) |
|  | Scored 7-9 | 37 (84.1) | 31 (96.9) | 197 (96.1) | 265 (94.3) |
| **Sepsis** | I don’t know OC | 4 (5.8) | 0 (0) | 7 (2.4) | 11 (2.7) |
|  | Scored 1-3 | 8 (11.6) | 4 (8.0) | 25 (8.7) | 37 (9.1) |
|  | Scored 7-9 | 50 (72.5) | 35 (70.0) | 201 (70.0) | 286 (70.4) |
| **Haemorrhagic shock** | I don’t know OC | 2 (2.9) | 2 (4.0) | 10 (3.5) | 14 (3.4) |
|  | Scored 1-3 | 5 (7.2) | 0 (0) | 8 (2.8) | 13 (3.2) |
|  | Scored 7-9 | 57 (82.6) | 48 (96.0) | 264 (92.0) | 369 (90.9) |
| **Admission to ITU** | I don’t know OC | 1 (2.3) | 1 (3.1) | 1 (0.5) | 3 (1.1) |
|  | Scored 1-3 | 3 (6.8) | 1 (3.1) | 10 (4.9) | 14 (5.0) |
|  | Scored 7-9 | 35 (79.5) | 25 (78.1) | 171 (83.4) | 231 (82.2) |
| **Mortality** | I don’t know OC | 1 (2.3) | 1 (3.1) | 3 (1.5) | 5 (1.8) |
|  | Scored 1-3 | 4 (9.1) | 2 (6.3) | 7 (3.4) | 13 (4.6) |
|  | Scored 7-9 | 39 (88.6) | 28 (87.5) | 189 (92.2) | 256 (91.1) |
| **Infertility** | I don’t know OC | 3 (4.3) | 0 (0) | 3 (1.0) | 6 (1.5) |
|  | Scored 1-3 | 9 (13.0) | 1 (2.0) | 12 (4.2) | 22 (5.4) |
|  | Scored 7-9 | 49 (71.0) | 44 (88.0) | 236 (82.2) | 329 (81.0) |
| **Normally sited (eutopic) subsequent pregnancy** | I don’t know OC | 2 (2.9) | 0 (0) | 2 (0.7) | 4 (1.0) |
|  | Scored 1-3 | 9 (13.0) | 1 (2.0) | 10 (3.5) | 20 (4.9) |
|  | Scored 7-9 | 51 (73.9) | 39 (78.0) | 240 (83.6) | 330 (81.3) |
| **Repeat CSEP** | I don’t know OC | 2 (2.9) | 1 (2.0) | 4 (1.4) | 7 (1.7) |
|  | Scored 1-3 | 9 (13.0) | 0 (0) | 9 (3.1) | 18 (4.4) |
|  | Scored 7-9 | 55 (79.7) | 43 (86.0) | 254 (88.5) | 352 (86.7) |
| **Treatment satisfaction** | I don’t know OC | 0 (0) | 0 (0) | 0 (0) | 0 (0) |
|  | Scored 1-3 | 2 (4.5) | 0 (0) | 2 (1.0) | 4 (1.4) |
|  | Scored 7-9 | 35 (79.5) | 25 (78.1) | 160 (78.0) | 220 (78.3) |

*Score 1-3 = 1- extremely unimportant, 2 - very unimportant, 3 – unimportant; Score 7-9 = 7 - important, 8 - very important, 9 - extremely important; OC, outcome.*
